# Melatonin: new insights on its therapeutic properties in diabetic complications

**DOI:** 10.1186/s13098-020-00537-z

**Published:** 2020-04-08

**Authors:** Mohammad Hossein Pourhanifeh, Azam Hosseinzadeh, Ehsan Dehdashtian, Karim Hemati, Saeed Mehrzadi

**Affiliations:** 1 Halal Research Center of IRI, FDA, Tehran, Iran; 2 Razi Drug Research Center, Iran University of Medical Sciences, Tehran, Iran; 3 School of Medicine, Iran University of Medical Sciences, Tehran, Iran; 4 Department of Anesthesiology, Iran University of Medical Sciences, Tehran, Iran

**Keywords:** Diabetes mellitus, Hyperglycemia, Melatonin, Cardiomyopathy, Neuropathy, Retinopathy, Nephropathy, Inflammation, Oxidative stress, Lipid metabolism

## Abstract

Diabetes and diabetic complications are considered as leading causes of both morbidity and mortality in the world. Unfortunately, routine medical treatments used for affected patients possess undesirable side effects, including kidney and liver damages as well as gastrointestinal adverse reactions. Therefore, exploring the novel therapeutic strategies for diabetic patients is a crucial issue. It has been recently shown that melatonin, as main product of the pineal gland, despite its various pharmacological features including anticancer, anti-aging, antioxidant and anti-inflammatory effects, exerts anti-diabetic properties through regulating various cellular mechanisms. The aim of the present review is to describe potential roles of melatonin in the treatment of diabetes and its complications.

## Background

Diabetes mellitus (DM), a frequent life-threatening metabolic disorder worldwide, is characterized by hyperglycemia caused by either insulin resistance or declined insulin secretion [1]. In the last decades, the incidence of diabetes mellitus has been enhancing [2, 3]. This disease has been one of the leading cause of death worldwide, and the International Diabetes Mellitus Federation estimated that 592 million people will suffer from diabetes mellitus by the year 2035 [4]. Several investigations have indicated that diabetes mellitus, could mediate various complications, including diabetic cardiovascular complications, diabetic neuropathy, retinopathy, nephropathy and liver complications, which have been the main causes of its mortality and morbidity [4, 5]. Thus, it is essential to search for efficient methods for the management and prevention of diabetes mellitus and its complications [6].

The impact of pineal hormone on insulin secretion, carbohydrate metabolism and blood glucose has been recently demonstrated. Melatonin (N-acetyl-5-methoxytryptamine), a tryptophan-derived endocrine agent, is mainly synthetized by the pineal gland and locally by numerous other tissues [7]. Despite the higher insulin levels in diabetic subjects, diabetes mellitus is accompanied by lower serum concentrations of melatonin in diabetic GotoKakizaki rats [8]. Furthermore, melatonin functions as an anti-aging [9], anti-inflammatory, antioxidant [10] as well as antihypertensive agent [11]. This review summarizes the therapeutic potentials of melatonin in the treatment of diabetes mellitus and its complications, based on findings of recent studies.

## Melatonin: potentials, safety and bioavailability

Melatonin, a broad spectrum antioxidant, can be used in various pathologic conditions mainly due to its regulatory effects on autophagy, endoplasmic reticulum (ER) stress, and oxidative stress. This molecule has potential therapeutic effects for treating neurodegenerative diseases, malignancies, cardiovascular diseases and many other clinical problems [12]. Of note, it also has a regulatory role in sleep disorders and reproduction [13, 14]. Furthermore, melatonin has immunomodulatory effect [15], which depends on its capability to increase the cytokines production and its anti-apoptotic and antioxidant properties. By affecting cytokine release from immunocompetent cells, melatonin has been suggested to modulate the immune system [16]. Melatonin modulates reproduction by regulating the production of sex hormones in seasonally breeding animals. In long-breeders, melatonin decreases estrogen production. However in short-breeders, melatonin increases the level of estrogen synthesis in winter [17, 18]. In humans, melatonin role on reproduction is not totally clear. During the dark season, the high level of melatonin is associated with the low estrogen production rate [19]. In addition, melatonin directly increases the steroidogenesis in human granulosa-luteal cells [20]. Furthermore, melatonin increases progesterone production via its M2 receptors on corpus luteum [21]. However, there are reports that indicate no role for melatonin in up-regulating estrogen synthesis by granulosa cells [22]. Taken together, melatonin affects the activity of ovaries; however this control and the exact mechanisms remain to be determined.

As mentioned earlier, melatonin is a very potent anti-oxidant partially due to its molecule structure which is both lipophilic and hydrophilic, so it can cross all bio-barriers easily and accumulate in high amounts within subcellular organelles including mitochondria which are the main sites for reactive oxygen species (ROS) production [23–26]. The anti-oxidative effects of melatonin is attributed to its direct free radical scavenging properties as well as inducing the production of other anti-oxidant enzymes including glutathione peroxidase (GPx), glutathione reductase (GR), catalase (CAT) and superoxide dismutase (SOD) [27–30]. Melatonin also inhibits the expression of pro-oxidant enzymes such as nitric oxide (NO) synthase (NOS), cyclooxygenase-2 (COX-2), myeloperoxidase and eosinophil peroxidase [31, 32]. Free radicals such as ROS are waste products of cellular metabolism. The accumulation of these agents within cells can cause damage to cell DNA, proteins and lipids and may give rise to cancerous cells. Melatonin can avoid these damages by neutralizing free radicals [30, 33, 34].

The safety of exogenous melatonin is still under investigation; however, with the limiting data available, melatonin can be considered as a safe drug. Melatonin administration for adolescents, children and preterm infants with different diseases has shown no significant side effects except at high doses or multiple time administration [35–37]. Of note, melatonin could affect sexual maturation in the mentioned groups [38]. In adults, oral administration of melatonin for improving of dyspnea (3 mg fast release for 3 months) in chronic obstructive pulmonary disease (COPD) patients and protecting the patients from depression and anxiety after breast cancer surgery (6 mg for 3 months) has shown no major side effects. However, minor adverse effects such as transient dizziness, headache, numbness, paresthesia of mouth, arms or legs, and deterioration of dyspnea has been considerable in oral administration [39, 40]. Melatonin use (9 mg sustained release per day for 4 weeks) in order to improve the sleep quality of the children with epilepsy had some adverse effects including: morning drowsiness, gastrointestinal symptoms, increased enuresis, headache, dizziness, diarrhea, rash, and hypothermia [41].

Since melatonin is mainly metabolized by cytochrome P450 (CYP) 1A2 and CYP2C19, inhibitors of these enzymes lead to higher concentration of melatonin in the body [42]. Melatonin lowers blood pressure and glucose level, therefore, patients receiving antihypertensive or blood glucose lowering drugs should use melatonin with cautious [43]. Since the administration of melatonin during pregnancy is not studied, its use is not recommended for pregnant women [38]. The use of melatonin by breastfeeding women causes daytime drowsiness for their infants [44].

Pharmacokinetics and optimal dosage of this therapeutic agent are not thoroughly clear yet and more studies are required on these subjects; however, there are some inconsistent studies on oral and intravenous administration of melatonin. The absorption of this hormone is site dependent making the rectum as the site with the highest absorption rate. Orally administered melatonin at doses up to 80 mg is absorbed fast and follows first-order kinetic [45]. Melatonin is expected to reach maximal plasma concentration after 30–45 min and 30–60 min (*t* max) in intravenous (IV) and oral administration, respectively [46, 47]. The elimination rate of melatonin from the body depends on the dose and route of administration. Because of structural characteristics, melatonin is both water and lipid soluble. So, it can run through tissues, cells and cellular compartments easily [48]. The elimination half-life (t1/2) for 100 mg melatonin in IV administration is 45 min [46], while it is 28–61 min for 0.005–2 mg [49]. The elimination half-life for oral administration of melatonin is a little higher and is about 46–65 min for 0.5–6 mg [47]. Melatonin is considered a drug with a high hepatic first pass effect. In oral administration, just 10–15% of melatonin reaches the systemic circulation and the rest of it is metabolized by CYP1A2 to 6-hydroxymelatonin and excreted in the urine after conjugation with sulfate or glucuronic acid [48, 50–53]. Of note, melatonin elimination occurs faster in children in comparison to adults [54]. Low absorption from the gastrointestinal tract, high first-pass effect and high rate of metabolizing make melatonin a drug with low bioavailability [53]. Because of poor bioavailability, other routes of administration are suggested; for example: subcutaneous injection, transdermal, oral transmucosal and intranasal [43, 55].

## Anti-diabetic effects of Melatonin

Melatonin has been shown to have a beneficial role in controlling blood glucose in both animal and human studies. The expression level of glucose transporter type 4 (GLUT4) gene is reduced in pinealectomized animals, which consequently results in glucose intolerance and insulin resistance. These conditions are alleviated by melatonin treatment [56, 57]. Furthermore, the level of melatonin decreases in human dental pulp tissue in type 2 diabetic participants. Melatonin in pharmacological concentration could improve iNOS and SOD activity in hyperglyceamic human dental pulp cells (hDPCs), suggesting the protective effects of melatonin in human dental pulp tissue under hyperglycaemia [58].  In rats with streptozotocin (STZ)-induced diabetes, eight-week treatment with insulin (NPH, 1.5 U/100gr/day) and melatonin (0.2 mg/kg/day in drinking water) improves glucose homeostasis and insulin sensitivity of white adipose tissue comparing with using one of these drugs individually [59]. The production of melatonin has been reported to reduce in animal models of diabetes and melatonin treatment (100 mg/kg/day in drinking water for 8 weeks) in high-fat diet-fed mouse with insulin resistance results in better glucose tolerance [60]. Diabetes also results in decreased testosterone production. It has been shown that melatonin (10 mcg/kg/day in drinking water) is able ameliorate the deleterious effects of diabetes on testosterone production in rats by improving glucose metabolism in the Leydig cells and inducing acetate production which is a precursor for cholesterol synthesis [61].

Patients with type 2 diabetes mellitus (T2DM) have a lower nocturnal melatonin production compared to ones without diabetes [62]. Several studies indicated that single dose of melatonin in healthy post-menopausal and pre-menopausal women (1 mg and 5 mg respectively) worsens the glucose tolerance test in the morning and the evening [63, 64]. On the other hand, chronic treatment with prolonged-release melatonin (2 mg) over a 5-month period decreases the level of HbA1c and betters glycemic control [65]. Furthermore, administration of melatonin (6 mg) for 3 months resulted in better glycemic control in patients with T2DM [66]. In patients with poorly controlled T2DM, addition of melatonin (10 mg) and zinc acetate (50 mg) to metformin resulted in the better tissue response to metformin alone [67]. In obese patients with Acanthosis Nigricans, melatonin (3 mg/day) supplementation for 12 weeks improved insulin sensitivity as well as inflammatory status [57].

Oxidative stress, which has an important role in the induction of various complications of diseases such as diabetes, is effectively attenuated by the anti-oxidative activity of melatonin. Moreover, this hormone protects the beta cells of pancreas, with low antioxidant content, by neutralizing reactive oxygen species [68, 69]. Based on a recent systematic review, melatonin may have a potential role in better glycemic control through increasing insulin sensitivity and lowering fasting glucose [70].

## Melatonin and diabetic complications

As mentioned above, diabetes mellitus causes numerous micro- and macrovascular complications in affected patients. Melatonin is considered as an appropriate candidate for the prevention and treatment of diabetes mellitus complications. Here, we discuss about its therapeutic potentials for cardiomyopathy, retinopathy, central nervous system (CNS)-related complications of diabetes, neuropathy, and nephropathy.

### Effects of melatonin on diabetic cardiomyopathy

Diabetic cardiomyopathy is a common complication of diabetes mellitus, which is defined as adverse changes in myocardial structure and function in the absence of other *cardiac* risk factors including coronary artery disease or hypertension [71]. Diabetic cardiomyopathy is characterized by increased myocardial fibrosis and stiffness, with this being associated with the impaired diastolic function, late systolic dysfunction resulting in the inability of the heart to pump enough blood through the body, a state called heart failure [72]. The main pathogenetic factors involved in the development and progression of diabetic cardiomyopathy include hyperglycemia, systemic insulin resistance, and impaired cardiac insulin metabolic signaling; these factors induce several pathways including vascular endothelial dysfunction, adrenergic activity, impairment of mitochondria calcium (Ca^2+^) handling, activation of renin–angiotensin system, myocardial ischemia/functional hypoxia, oxidative stress, mitochondria dysfunction, inflammation, endoplasmic reticulum stress and cardiomyocyte death [72, 73]. Excessive level of reactive oxygen species (ROS) induced by high glucose contributes to the peroxidation of lipids, induction of apoptosis and inhibition of autophagy in muscle fibers [74]; diabetes impairs myocardial mitochondrial biogenesis and suppresses the activity of myocardial mitochondrial Complexes I, III and IV resulting in the loss of mitochondrial number, impairment of mitochondrial function, increased generation of ROS and escape of death‐inducing factors [75]. Although hyperglycemia increases the risk of cardiomyopathy in diabetic patients and good glycemic control lowers the progression of diabetic microvascular complications, intensive glucose control in patients with established diabetes mellitus cannot reduce the mortality induced by diabetic cardiomyopathy [76]. This highlights the need for effective therapeutic agents to prevent or treat diabetic cardiomyopathy.

**Fig. 1:**
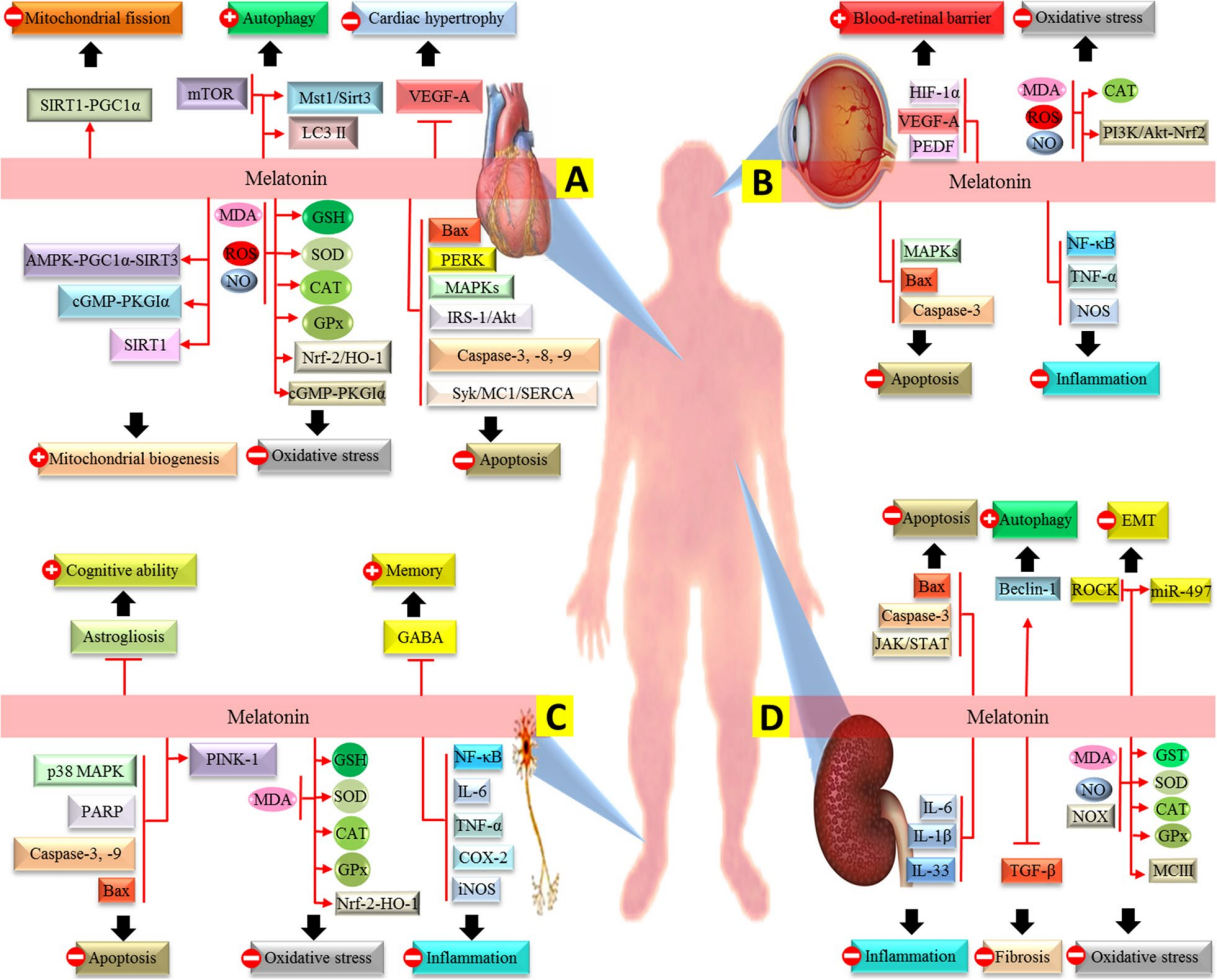
**a** Melatonin improves diabetic cardiomyopathy through inhibiting mitochondrial fission by activation of SIRT1‐PGC1α pathway, increasing mitochondrial biogenesis by activation of cGMP-PKGIα, SIRT1 and AMPK-PGC1α-SIRT3 signaling pathways, inhibiting cardiac hypertrophy by reduction of the expression of VEGF-A, inhibiting apoptotic pathway by decrease the expression of caspase-3, -9, -8, Bax, PERK, Syk/MC1/SERCA, IRS-1/Akt and MAPK signaling pathways and inhibiting oxidative stress by increase the activity of SOD, CAT, GPx, cGMP-PKGIα and Nrf-2-HO-1 signaling pathways and the level of GSH and reduction of ROS, MDA and NO levels. **b** Melatonin reduces diabetic retinopathy through inhibiting oxidative stress by reduction of ROS, MDA and NO levels and activation of CAT and PI3K/Akt-Nrf2 pathway, improving blood-retinal barrier by reduction of the expression of HIF-1α, VEGF-A and PEDF, inhibiting apoptotic pathway by decrease the expression of caspase-3, Bax and MAPKs pathways, and inhibiting inflammation by inhibition of the expression of TNF-α, NOS and the activity of NFκB. **c** Melatonin ameliorates diabetic neuropathy through inhibiting oxidative stress by increasing CAT, SOD and GPx activity and GSH level and activating Nrf-2-HO-1 pathway, inhibiting inflammation by reduction of TNF-α, iNOS, IL-6 and COX-2 expressions and the activity of NFκB, inhibiting apoptotic pathway by alleviation of the expression of caspase-3 and -9, Bax, PARP, and p38 MAPKs and elevation of PINK-1 level, and increasing the level of GABA and decreasing astrogliosis, which this effect leads to the improvement of memory and cognitive ability. **d** Melatonin improves diabetic nephropathy through inhibiting fibrotic process by reduction of the expression of TGF-β, inhibiting oxidative stress by enhancement of mitochondrial complex III, CAT, SOD, GPx and GST activities and reduction of NOX activity and MDA and NO generation, inhibiting inflammation by decreasing the level of IL-β, IL-6 and IL-33, inhibiting apoptosis by reduction of caspase-3 and Bax expression and JAK/STAT activity, inducing autophagy pathway by enhancement of the expression of Beclin-1, and inhibiting EMT by elevation the level of miR-49, which results in the alleviation of the level of ROCK. *SIRT* sirtuin, *PGC1α* peroxisome proliferator-activated receptor gamma coactivator 1α, *AMPK* 5′ AMP-activated protein kinase, *cGMP* Cyclic guanosine monophosphate, *PKGIα* Protein kinase G Iα, *VEGF-A* Vascular endothelial growth factor-A, *Syk* Spleen tyrosine kinase, *SERCA* sarcoendoplasmic reticulum calcium transport ATPase, *Nrf2* erythroid 2‐related factor 2, *HO-1* heme oxygenase-1, *IRS‐1* insulin receptor substrate, *Akt* Protein kinase B, *GPx* glutathione peroxidase, *CAT* catalase, *SOD* superoxide dismutase, *NOS* NO synthase, *iNOS* inducible NOS, *MDA* malondialdehyde, *COX-2* cyclooxygenase-2, *ROS* reactive oxygen species, *NF‐κB* nuclear factor-κB, *TNF-α* tumor necrosis factor α, *IL* interleukin, *mTOR* mammalian target of rapamycin, *GSH* glutathione, *NO* nitric oxide, *JAK* janus kinase, *STAT* signal transducer and activator of transcription, *GABA* gamma-aminobutyric acid, *PARP* poly(ADP-ribose) polymerase, *MAPK* mitogen-activated protein kinase, *TGF-β* transforming growth factor-β, *EMT* endothelial-to-mesenchymal transition, *ROCK* RhoA/Rho kinase, *GST* glutathione *S*-transferases, *NOX* NADPH oxidase

Melatonin as an effective antioxidant has been demonstrated to prevent diabetes-induced harmful effects on the heart (Fig. 1a). Melatonin reduces oxidative damage in myocardial cells and inhibits extrinsic and intrinsic pathways of apoptosis; in STZ‐induced diabetic rats, melatonin improves antioxidative status and reduces lipid peroxidation level and apoptotic markers in heart tissue to near control values [77, 78]. Melatonin preserves mitochondrial function in cardiac of diabetic rats through increasing the mitochondrial biogenesis and deacetylation of mitochondrial anti-oxidative enzymes, which this effect results from activation of cGMP-PKGIα, sirtuin-1 (SIRT1)‐peroxisome proliferator-activated receptor gamma coactivator 1α (PGC1α) and 5′ AMP-activated protein kinase (AMPK)-PGC1α-SIRT3 signaling pathways [79–81]. Activation of PGC1α-SIRT3 signaling plays a key role in cardioprotective action of melatonin; this signaling pathway leads to the enhancement of mitochondrial SOD activity, oxidative phosphorylation of Complexes I, III and IV, and reduction of mitochondrial lipid peroxidation, ROS generation and myocardial apoptosis [79]. In addition, it has been emphasized that melatonin treatment ameliorates myocardial apoptosis through suppressing ER-stress and spleen tyrosine kinase (Syk)/mitochondrial complex I/sarcoendoplasmic reticulum calcium transport ATPase (SERCA) axis [82, 83]. Chronic high-glucose activates Syk leading to the repression of the expression and activity of mitochondrial complex I causing increased generation of ROS and subsequent peroxidation of SERCA; this results in the inhibition of cytoplasmic calcium re-uptake by ER leading to cellular calcium overload and cardiomyocytes death via activation of mitochondria‐ and ER stress‐mediated apoptosis [82, 83].

Melatonin modulates nuclear factor erythroid 2-related factor 2 (NRF2)-hemeoxygenase 1 (HO-1) and mitogen-activated protein kinase (MAPK) signaling through activating membrane receptors (especially MT2 receptor)-dependent cyclic guanosine monophosphate (cGMP)-protein kinase G Iα (PKGIα) signaling pathway, which contributes to the improvement of diabetic cardiac function by inhibiting myocardial apoptosis and oxidative stress [80]. Activation of SIRT1‐PGC1α pathway by melatonin contributes to the inhibition of dynamin‐related protein 1 (Drp1)‐mediated mitochondrial fission, suppression of oxidative stress, reduction of cardiomyocyte apoptosis, and improvement of mitochondrial and cardiac function in STZ‐induced diabetic mice [81]. Furthermore, maternal melatonin treatment improves the tolerance to myocardial ischemia/reperfusion injury in diabetic mother–offspring mice. This beneficial effect of melatonin results from activation of cardiac insulin receptor substrate (IRS)‐1/Protein kinase B (Akt) signaling leading to the suppression of mitochondria‐ and ER stress-mediated apoptosis and oxidative stress [84]. Co-administration of PPAR-g agonist, thiazolidinediones, with melatonin has been reported to have beneficial effects on diabetes-induced cardiovascular complications. Combination of pioglitazone or rosiglitazone with melatonin is effective to normalize levels of SOD, glutathione (GSH), CAT, and lipid peroxidation as well as inhibit myonecrosis, vacuolar changes and infiltration of inflammatory cells in the heart tissue of alloxan-induced diabetic rats; this indicates that melatonin may be a potential drug to increase beneficial effect of thiazolidinediones on diabetic cardiomyopathy [73]. Melatonin treatment may also prevent the development of diabetic cardiomyopathy through increasing the phosphorylation of vascular endothelial growth factor-A (VEGF-A) resulting in the inhibition of cardiac enlargement and hypertrophy in STZ-induced diabetic rats [85].

The protective effect of melatonin against diabetic cardiomyopathy may also result from its modulatory effect on autophagy pathway. Melatonin has been reported to alleviate cardiac remodeling through inhibiting the expression of mammalian target of rapamycin (mTOR); mTOR is an important molecule inhibiting autophagy pathway, which remarkably elevates in the myocardium of diabetic rats leading to the occurrence of both cardiomyopathy and cardiac hypertrophy [74]. Melatonin also regulates the activity of mammalian Ste20-like kinase 1 (Mst1)/Sirt3 signaling pathway. Suppression of Mst1 by melatonin contributes to the up-regulation of autophagy/Parkin-mediated mitophagy, inhibition of apoptosis and modulation of mitochondrial integrity and biogenesis leading to the alleviation of cardiac remodeling and dysfunction in diabetic mice [86, 87]. Table 1 summarizes studies indicating the protective effects of melatonin on diabetic cardiomyopathy.

**Table 1:** Investigations on melatonin and diabetic cardiomyopathy

Type of study		Route of melatonin administration	Treatment duration	Target	Effect	Refs.
In vivo dose (animal)	In vitro concentration (cell type)					
50 mg/kg/day (male Wistar rats)	–	Intraperitoneal	56 days	mTOR signaling SOD CAT GPx	Anti-hyperglycemic and antioxidant effects	[74]
50 mg/kg/day (male Wistar rats)	–	Intraperitoneal	56 days	VEGF-A	Antioxidant effects Preventive effects on cardiac hypertrophy	[85]
20 mg/kg/day (Parkin^−/−^ mice)	–	Oral	4 weeks	Mst1, LC3 II	Up-regulated mitophagy	[86]
20 mg/kg/day (Syk^CKO^mice)	–	ND	12 weeks	Syk/MC1/SERCA pathway	Antioxidant effects Anti-apoptotic effects	[82]
10 mg/kg/day SIRT1^−/−^ mice	100 μmol/L (cardiomyocytes)	Intraperitoneal	10 weeks	SIRT1‐PGC1α pathway	Preventive effects on mitochondrial fission	[81]
10 mg/kg/day (male Sprague–Dawley rats)	–	Oral	24 weeks	PERK, Bcl-2, Bax, Caspase-3	Anti-apoptotic effects Anti-ER stress effects	[83]
20 mg/kg/day (C57BL/6 wild type mice)	100 μM (cardiomyocytes)	Oral	4 weeks	Mst1/Sirt3, LC3-II	Up-regulated autophagy Anti-apoptotic effects	[87]
10 mg/kg/day (male Wistar rats)	–	Oral	21 days	Caspase-3, Caspase-8, Caspase-9	Antioxidant effects	[78]
10 mg/kg/day (female Wistar strain rats)	–	Intraperitoneal	6 weeks	MDA GSH SOD	Antioxidant effects	[77]
10 mg/kg/day (male Sprague–Dawley rats)	10 μmol/l (H9c2)	Oral (for 5 days before the surgery) Intraperitoneal (once 10 min before the reperfusion)	5 days	AMPK-PGC-1α-SIRT3 signaling	Ameliorative effects on myocardial ischemia–reperfusion	[79]
10 mg/kg/day (male Sprague–Dawley rats)	10 μM (H9c2)	Oral (for 5 days before the surgery) Intraperitoneal (once 10 min before the reperfusion)	5 days	cGMP-PKGIα, Nrf-2-HO-1, and MAPK signaling pathways	Ameliorative effects on myocardial ischemia–reperfusion Anti-apoptosis effects Antioxidant effects	[80]
10 mg/kg/day (female C57BL/6 mice)	–	Intraperitoneal	Until the end of gestation	IRS-1/Akt signaling	Ameliorative effects on myocardial ischemia–reperfusion in diabetic pregnancy	[84]

### The therapeutic effects of melatonin on diabetic retinopathy

Diabetic retinopathy (DR), a prevalent asymptomatic microvascular complication of diabetes mellitus, is a leading cause of vision loss worldwide [88, 89]. According to recent reports, one-third of diabetic patients possess some degree of diabetic retinopathy [90]. Due to the increasing prevalence of diabetes worldwide, the number of diabetic retinopathy patients has been estimated to elevate from 424.9 million in 2017 to 628 million by 2045 [91].

Nowadays, as mentioned earlier, inflammation and oxidative stress have crucial roles in diabetic retinopathy pathogenesis [92, 93]. Therefore, treatments such as melatonin restricting inflammatory and oxidative impacts of diabetes mellitus could be greatly potential to affect individuals through preventing or decreasing the retinal complications (Fig. 1b).

Various and heterogeneous factors, such as advanced glycation end products (AGEs), growth factors, hyperglycaemia as well as great levels of vitreous or circulating chemokines, cytokines and ROS can trigger the inflammatory responses in the retinal vasculature [94, 95]. Thus, mounting evidence suggests chronic inflammation as an essential process in the development of diabetic retinopathy, primarily in early stages [96].

Several investigations have shown enhanced levels of different pro-inflammatory cytokines, including COX2, inducible NO synthase (iNOS), nuclear factor kappa B (NF-*κ*B), intercellular adhesion molecule-1 (ICAM), and vascular endothelial growth factor (VEGF) in the retinas and vitreous humours of diabetic animals and humans [92, 97, 98]. Although various stimuli are able to promote the production of VEGF leading to pathological angiogenesis during retinal hypoxia [99], oxidative stress has a key role via enhancing the level of growth factor and mediating the evolution of diabetic retinopathy to pathological advanced stages. Utilizing retinal pigment epithelial cells, Simão and colleagues showed that high levels of glucose regulate the expression of different VEGF isoforms through ROS generation [100].

Melatonin synthesis has been reported to reduce in diabetic rats, which this is characterized by the arylalkylamine *N*‐acetyl transferase (AANAT) activity reduction, as a significant enzyme regulating melatonin daily production; insulin therapy has been reported to ameliorate decreased melatonin levels [101, 102]. In agreement with these findings, Hikichi et al. also reported that nighttime levels of melatonin were considerably lower in diabetic patients compared to non-diabetic subjects, and these levels were lower in patients with proliferative diabetic retinopathy than non-proliferative diabetic retinopathy individuals [103]. In diabetic rats, melatonin reduces histopathological alterations of the retina through decreasing oxidative stress [104]. Dehdashtian et al. [30] reviewed the involvement of inflammation, oxidative stress and autophagy in the pathogenesis of diabetic retinopathy. They indicated that melatonin is able to modulate autophagy cascade and diminishes oxidative stress and inflammation. Melatonin enhances cellular antioxidant defenses and reduces the production of VEGF in Müller cells through activating the PI3K/Akt-Nrf2 signaling pathway; these anti-angiogenic and anti-oxidative effects of melatonin in Müller cells have shown to be receptor-dependent [105]. Melatonin treatment triggers Akt-Nrf2-induced antioxidant system and suppresses NF-*κ*B pathway; it is suggested that regulating retinal melatonin and its receptors could be a beneficial method for treatment of diabetic retinopathy [106]. Melatonin reduces the mean scores of fluorescein leakage and the level of ROS and malondialdehyde (MDA) as well as suppresses the apoptosis and inflammation in rats with diabetic retinopathy through repressing the MAPK pathway [107, 108].

Djordjevic et al. [109] investigated the protective effects of melatonin on retina in pre-diabetic conditions. They showed that oral administration of melatonin (85 μg/animal/day) increases HDL cholesterol concentrations in treated rats and decreases fructosamine and fasting serum glucose levels. In addition, it affected quantitative insulin sensitivity check index and serum insulin in treated groups but had no considerable effects on non-fasting glucose. Totally, melatonin supplementation significantly diminished matrix metallopeptidase 9 (MMP9), VEGF, iNOS, advanced oxidation protein products (AOPP), and MDA levels, hence showing overall positive antioxidant impacts as well as pro-angiogenic signaling in the pre-diabetic retina. Ma and his colleagues investigated the therapeutic effects of melatonin on diabetic retinopathy rats. They showed that the expression of Bax, a pro-apoptotic gene, significantly increases in the retina of diabetic retinopathy rats, while the expression of Bcl-2, an anti-apoptosis gene, decreases. After melatonin therapy, the serum levels of interleukin (IL)-1β and IL-18 also reduces. These results showed that melatonin has anti-inflammatory and anti-apoptosis effects in rats with diabetic retinopathy [107]. Ozdemir et al. [110] showed that daily intraperitoneal administration of melatonin for 12 weeks declines retinal levels of MDA and nitrotyrosine, indicating an antioxidant support. Melatonin also normalizes retinal vascular alterations through decreasing the vasculoregulator cytokines. Studies reporting melatonin effects on diabetic retinopathy have been illustrated in Table 2.

**Table 2:** Effects of melatonin on diabetic retinopathy reported by various investigations

Type of study		Route of administration	Treatment duration	Target	Effect(s)	Refs.
In vivo dose (animal)	In vitro concentration (cell type)					
10 mg/kg/day (male Sprague–Dawley rats)	–	Intraperitoneal	7 days	MAPK pathway, Bcl-2, Bax, Caspase-3	Anti-apoptotic effects, Anti-inflammatory effects	[107]
10 mg/kg/day (male Wistar rats)	–	Intraperitoneal	4 weeks	HIF-1*α*, VEGF-A, PEDF	Antioxidant effects	[110]
20 mg (male Wistar rats)	–	Subcutaneous pellet	12 weeks	NOS, TNF-α, CAT,	Protective effects on the retina against the alterations	[196]
20 mg/kg/day (male WISTAR rats)	–	Oral	7 weeks	MDA, ROS	Protective effects on the retina against the alterations	[108]
10 mg/kg/day (male Sprague–Dawley rats)	–	Intraperitoneal	12 weeks	glutamate cysteine ligase (GCL), Nrf2, TNF-α, iNOS, NF-κB	Anti-inflammatory effects, Antioxidant effects	[106]
–	10 nM–0.1 mM (Müller cells)	–	48 h	VEGF, Akt	Antioxidant effects	[105]

### The therapeutic effects of melatonin on diabetic neuronal complications

#### Melatonin and CNS-related complications of diabetes

##### Cognitive functions

Acute and chronic vascular and metabolic perturbations impair the structural and functional brain integrity in diabetic patients. In other words, DM contributes to various structural and functional disorders related to peripheral and central nervous systems [111].

Cerebrovascular alterations, such as neurotropic changes like decreased IGF (insulin growth factor) levels, disrupted vascular reactivity, and attenuated blood flow to the brain demonstrate that mentioned aberrations precede apoptotic neuronal loss as well as functional cognitive disturbances in hippocampus [112, 113]. DM is correlated with moderate impairment in cognitive functions, Alzheimer’s disease and dementia [114–117]. Cognitive deficit is probably linked to hyperglycemia neurotoxic impacts and alterations in neuronal and neurotransmission functionalities. Enhanced oxidative stress promotes oxidative damages in multiple brain regions, such as the hippocampus [118]. As mentioned above, oxidative stress plays key contributory roles in the development of diabetic complications, while antioxidant treatment may restore or protect physiological functions [118]. Prior researchers have reported that antioxidants decrease the hippocampal neuronal cell damages induced by diabetes-mediated excitotoxicity [119–121]. It has been revealed that free radical scavengers protect neurons from diverse neurodegenerative situations. Melatonin, by its antioxidant ability, has been indicated to exert neuroprotective effects under different circumstances [122, 123].

In a study, Tuzuc and colleagues examined and compared the vitamin E and melatonin impacts on cognitive function of diabetic animals. Memory and learning behaviors were studies utilizing a spatial version of the Morris water maze test. Glutathione and lipid peroxidation levels were measured in frontal cortex and hippocampus. The diabetic animals developed considerable impairments in memory and learning behaviors in comparison with control rats. Moreover, the concentration of glutathione is reduced and the level of lipid peroxidation is enhanced in diabetic animals. Vitamin E and melatonin remarkably improved memory and learning performance. In addition, glutathione levels and lipid peroxidation were reversed by these nutraceuticals toward the control values. It can be concluded that, in diabetic patients, oxidative stress contributes to memory and learning deficits, suggesting that antioxidants including vitamin E and melatonin are able to ameliorate cognitive impairments in diabetes [124].

Administration of melatonin in STZ-induced diabetic rats with elevated level of glial fibrillary acidic protein (GFAP) and S100B, as an indicator of astrogliosis, prevents lipid peroxidation, GFAP and and S100B synthesis and the loss of neural cell adhesion molecule, with these effects being associated with the improvement of cognitive ability [125, 126]. Melatonin may show these effects through its antioxidant activity contributing to the increased level of brain antioxidant, decreased brain NOSs activities and plasma cytokines levels, and inhibition of astrocytes apoptosis playing an important role in the CNS homeostasis [104, 127, 128]. Inhibition of oxidative stress-induced activation of Poly(ADP-ribose) polymerase (PARP) cascade is another mechanism by which melatonin can improve high glucose-induced changes in neurotransmitter (glutamate and gamma-Aminobutyric acid (GABA)) levels and neurobehavioral parameters in diabetic rats with neuropathy; over-activation of PARP in response to ROS-induced DNA damage initiates an energy-consuming cycle leading to the depletion of intracellular NAD^+^ and ATP culminating into energy failure and cell death [129, 130]. A 2-week combination treatment with nicotinamide (300, 1000 mg/kg/day) and melatonin (3, 10 mg/kg/day), or their application as single therapeutic agents ameliorated the neurobehavioral parameters, and GABA and glutamate levels. In general, they showed that concurrent suppression of oxidative stress-PARP overactivation pathway can be valuable in the therapy of DM-related CNS disorders [130].

##### Depression

DM patients are approximately three times more probable to develop depression [131, 132]. Even pregnant females with gestational diabetes have greater incidence of pregnancy-related depression [133, 134]. Similar to other complications, hyperglycemia is correlated with depression [135], and glycemic therapy reduces depression risk even postpartum depression [136, 137]. Antioxidant defense breakdown may occur in chronic depression, due to a promoted ROS production and an attenuated antioxidant system activity [138].

Regarding antioxidant properties of melatonin, Ergenc and co-workers examined melatonin effects on S100B, AGE receptor (AGER), and AGE levels, oxidative stress, and anxiety and depressive like-behaviors in streptozotocin-mediated diabetic rats. They showed that, in addition to the amelioration of anxiety and depressive like-behaviors, melatonin reversed DM-mediated reduction of S100B and GSH, and enhancement of AGE and MDA levels, indicating anxiolytic and antidepressant potentials in diabetic rats via normalizing oxidative stress, S100B, and AGE/AGER in the hippocampus and prefrontal cortex [69].

Furthermore, Rebai et al. also elucidated that melatonin and fluoxetine decreased anxiety and depression signs, and Hemoglobin A1C (HbA1c) levels in diabetic rats. Melatonin and fluoxetine diminished thiobarbituric acid reactive substances in both cortices. The activity of CAT was restored by melatonin and fluoxetine, while only melatonin ameliorated GPx and glutathione S-transferases (GST) activity in the prefrontal cortex. Moreover, melatonin restored GPx activity in the hippocampus, while fluoxetine had no impact. Overall, they illustrated that antioxidants and antidepressants can counter oxidative disorders and mood related to DM [139]. Altogether, melatonin is a promising therapeutic agent for the treatment of CNS-related complications of DM and further studies are needed to be carried out to prove its efficacy and find possible actions mechanisms as well. Table 3 summarizes investigations evaluating the effect of melatonin on CNS-related complications of diabetes.

**Table 3:** Studies of melatonin effects on CNS-related complications of diabetes

Type of study		Route of administration	Treatment duration	Target	Effect(s)	Refs.
In vivo dose (animal)	In vitro concentration (cell type)					
10 mg/kg/day (male Wistar rats)	–	Intraperitoneal	2 weeks	Total antioxidant status, GSH, GPx, lipid peroxidation, IL-1β and IL-4	Antioxidant effects	[128]
10 mg/kg/day (male Wistar rats)	–	Intraperitoneal	7 days	NCAM GFAP	Prevented cognitive impairments Antioxidant effects	[125]
10 mg/kg/day (Male Wistar rats)	–	Intraperitoneal	7 weeks	GSH, lipid peroxidation,	Improved memory and learning performance	[124]
10 mg/kg/day (male Wistar rats)	–	Intraperitoneal	6 weeks	GFAP S100B MDA GSH	Reduced glial reactivity in the cerebellum, cortex, and hippocampus Antioxidant effects	[126]
1 mg/kg/day (Male albino rattus norvegicus rats)	–	Intraperitoneal	4 weeks	GSH, SOD, CAT, lipid peroxidation	Anti-apoptotic effects Antioxidant effects	[127]
10 mg/kg/day (male Wistar rats)	–	Intraperitoneal	2 weeks	NOS	Anti-apoptotic effects Antioxidant effects	[104]
3, 10 mg/kg/day (male Sprague–Dawley rats)	–	Oral	2 weeks	GABA-glutamate homeostasisOxidative stress-PARP pathway	Improved neurochemical and neurobehavioral changes	[130]
10 mg/kg/day (male Wistar rats)	–	Intraperitoneal	4 weeks	S100B and GSH, and enhancement of AGE, lipid peroxidation	Ameliorated anxiety and depressive like-behaviors	[69]
10 mg/kg/day (male Wistar rats)	–	Intraperitoneal	4 weeks	GST, CAT, GPx, lipid peroxidation	Decreased anxiety and depression signs, and HbA1c levels	[139]

### Therapeutic potentials of melatonin for diabetic neuropathy

Neuropathy is a common complication of diabetes mellitus which approximately affects life quality of 50% of diabetic patients [140]. Oxidative stress and inflammation induced by hyperglycemia plays an important role in the pathogenesis of diabetic neuropathy [141]. The high incidence of diabetic neuropathy is due to the high oxygen requirement of the brain, high content of polyunsaturated fatty acids (PUFAs) and inefficient antioxidant capacity of the brain tissue, which results in the increased vulnerability of brain to diabetes-induced oxidative stress [128]. Prolonged hyperglycaemia promotes hippocampal and cortical neuron apoptosis in diabetic patients leading to the moderate impairments in cognitive function and neurodegenerative diseases such as Alzheimer’s disease [142]. Diabetes mellitus also damages somatic or autonomic nerves causing neuropathic pain, which contributes to the disruption of patients’ daily functions and reduction of life quality [143]. Due to the role of oxidative stress in the induction of diabetic neuropathy, administration of antioxidants such as melatonin may be a therapeutic approach to prevention of diabetic neuropathy (Fig. 1c).

Melatonin secretory patterns is disturbed in diabetic patients with autonomic neuropathy indicating a complex interaction between melatonin dynamics and autonomous neuropathy [144, 145]. Administration of melatonin has been reported to prevent hyperglycemia-induced neurotic damage and occurrence of diabetic neuropathy characterized by increasing motor nerve conduction velocity (MNCV) and sciatic nerve diameter as well as reduction of thermal hyperalgesia [146, 147]. The antihyperalgesic effect of melatonin is suggested to be mediated by the inhibition of l-arginine–NO pathway and induction of opioidergic system [148]. Furthermore, the analgesic properties of melatonin have been reported to results from it activity on melatonin MT2 receptor through modulation of brainstem descending antinociceptive pathways [149, 150]. In dorsal root ganglia neurons of mice, the analgesic effects of melatonin cause from MT2-dependent and independent pathways. The MT2-dependent pathway mediates by the activation of RAR-related orphan receptor alpha (RORα) leading to the inhibition of MAPK1 and subsequent calcium signaling pathways; this effect results in the c-fos, calcitonin gene-related peptide (CGRP), and neuro-inflammatory cytokines such as tumor necrosis factor (TNF)-α and IL-1β. The MT2-independent pathway mediates by inhibition of the expression of nitric oxide synthase 1 (NOS1) [151].

As mentioned previously, high glucose induces oxidative stress and increases Ca^2+^ ion entry playing important roles in the pathogenesis of diabetic neuropathy [152]. Treatment with melatonin improves hippocampal injury and peripheral neuropathic pain in STZ-induced diabetic rats; these protective activities of melatonin are characterized by the improvement in the spatial navigation memory and results from electrophysiological studies including tibial motor nerve conduction and cortical tibial nerve somatosensory evoked potentials [153–155]. Melatonin inhibits oxidative stress-induced Ca^2+^ mobilization through transient receptor potential (TRP) melastatin 2 (TRPM2) and TRP vanilloid type 1 (TRPV1) channels resulting in the prevention of mitochondrial depolarization, intracellular ROS production and apoptosis in neurons [153]. Histopathological, immunohistochemical and ultrastructural studies also have confirmed the neuroprotective effect of melatonin in STZ-induced diabetic rats with peripheral and central neuropathy. In the sciatic nerves, melatonin has been reported to reduce vacuolization of the myelin sheath, axon–myelin separation and the number of degenerated fibers. In cerebral cortices, melatonin-treated diabetic rats showed the reduction in the neurodegenerative and necrotic changes; spongiosis and reactive astrogliosis were absent and scattered pyknotic neurons with perineural vacuolations and swollen neurons with karolysis were remarkably reduced in animals with cerebral diabetic neuropathy [156, 157].

The modulatory effect of melatonin on inflammatory and autophagy pathways is considered as an important mechanism against diabetic neuropathy. Melatonin elevates the expression of nuclear factor erythroid 2‐related factor 2 (Nrf2) resulting in the up-regulation of the expression of phase II antioxidant/detoxifying enzymes such as heme oxygenase-1 (HO-1) decreasing oxidative stress and activation of nuclear factor-κB (NF‐κB) cascade [158].The expression of PINK1 and microtubule-associated proteins 1A/1B light chain 3 B (LC-3 B), mitophagy markers, increased in neuronal cells following treatment with melatonin. Since silencing of PINK1 expression impairs melatonin-mediated reduction of mitochondrial ROS production and apoptotic marker activation, it is suggested that melatonin prevents neuronal cell apoptosis under high glucose conditions through stimulating PINK1 expression; this effect of melatonin has been demonstrated to be MT2 receptor dependent [159].

Erectile dysfunction is an intractable health problem impressing more than half of male diabetic patients. Evaluation of the effect of melatonin on erectile dysfunction in diabetic rats revealed that melatonin treatment improves the erectile function through ameliorating neuropathy in dorsal penile nerve and major pelvic ganglion. This effect of melatonin is accompanied by alleviation of fibrosis through reduction of collagen deposition, oxidative stress, and p38 MAPK signaling pathway in penis [160]. Retina neuronal cell apoptosis is one of the main pathological symptoms of diabetic retinal neuropathy, which induces by the high glucose-induced excessive production of ROS resulting in the structure and function damages of mitochondria triggering the release of apoptosis-inducing factors and subsequent activation of neuronal apoptosis process [161]. Table 4 summarizes in vivo and in vitro studies evaluating the effect of melatonin on diabetic neuropathy.

**Table 4:** Studies of melatonin effects on diabetic neuropathy

Type of study		Route of administration	Treatment duration	Target	Effect(s)	Refs.
In vivo dose (animal)	In vitro concentration (cell type)					
50 mg/kg/day (male Wistar rats)	–	Intraperitoneal	45 days	SOD CAT GPx MDA	Improved neurodegeneration and showed antioxidant effects	[156]
10 mg/kg/day (male Sprague–Dawley rats)	–	Intraperitoneal	4 weeks	p38 MAPK signaling	Ameliorated erectile function and improved fibrosis and neuropathy, Antioxidant effects	[160]
10 mg/kg/day (male Wistar albino rats)	–	Intraperitoneal	2 weeks	–	Increased tibial nerve conduction velocity and amplitude	[154]
10 mg/kg/day (male Wistar albino rats)	–	Intraperitoneal	6 weeks	–	Increased MNCV and sciatic nerve diameter	[146]
120 mg/kg/day (Wistar–Kyoto rats)	–	Intraperitoneal	1 week	–	Decreased thermal hyperalgesia	[147]
10 mg/kg/day (male Sprague–Dawley rats)	–	Intraperitoneal	12 weeks	Caspase-3, Mn SOD, Cu–Zn SOD	Anti-apoptotic and antioxidant effects	[161]
3, 10 mg/kg/day (male Sprague–Dawley rats)	–	Oral	2 weeks (seventh and eighth week after diabetes induction)	TNF-α, IL-6, iNOS, COX-2, Nrf2, HO-1	Anti-inflammatory and antioxidant effects	[158]
3, 10 mg/kg/day (male Sprague–Dawley rats)	–	Oral	2 weeks	MDA NAD^+^, ATP, PARP	Improved functional deficits and pain parameters Antioxidant effects	[129]
10 mg/kg/day (female Wistar albino rats)	–	Intraperitoneal	2 weeks	TRPV1 and TRPM2 channels Caspase-3 Caspase-9	Neuroprotective activity Antioxidant effects Anti-apoptosis effects	[153]
10 mg/kg/day (male Wistar rats)	–	Intraperitoneal	2 weeks	GSH-Px CAT Lipid peroxidation	Improved spatial navigation memory Antioxidant effects	[155]
10 mg/kg/day (male albino rats)	–	Intraperitoneal	6 weeks	Myelin sheaths, nerve fibers and endoneurium in sciatic nerve sections	Antioxidant and hypoglycemic effects Myelin sheath vacuolization reduced Mild local axon separation from myelin sheaths was detected	[157]
–	1 μmol/L (SK‐N‐MC, SH‐SY5Y)	–	24 h	MT_2_/Akt/NF-κB pathway Caspase-3 Caspase-9 PINK1	Anti-apoptotic effects Antioxidant effects	[159]

### Melatonin is an appropriate agent for the treatment of diabetic nephropathy

Diabetic nephropathy, as one of the main microvascular complications of both type 1 and type 2 diabetes mellitus, is the most frequent cause of end-stage renal diseases. Diabetic nephropathy is characterized by nephron enlargement, glomerular hyperfiltration as well as the hypertrophy of mesangial cells, which eventually develops into glomerulosclerosis [162]. Several mechanisms have been proposed for diabetic nephropathy progression and development, including lipid disorders, oxidative stress, pro-fibrotic and fibrotic cytokines generation (such as fibronectin (FN)-1, plasminogen activator inhibitor (PAI)-1, and connective tissue growth factor (CTGF) [163–166].

As mentioned above, ROS have a key role in diabetic complications [167]. In hyperglycemia state, the persistent oxidative stress contributes to damage to the nuclear DNA and the mitochondrial genetic material [168].

Hyperglycemia leads to apoptosis in different types of cells in diabetic nephropathy, such as the proximal tubule epithelial cells [169]. It has been previously explained that the apoptosis occurs through activating numerous intracellular signaling pathway [170]. Now, it is acknowledged that hyperglycemia mediates apoptosis and promotes the gradual loss of kidney function in diabetic nephropathy [171].

Structural melatonin’s features, such as electron-richness, hydrophilicity, and lipophilicity yield potential impacts of this agent in terms of antioxidant capacity and insulin secretion impact by the MT1 receptor [172]. Melatonin elevates numerous antioxidant enzymes, such as SOD, peroxidase, GSH and lipid peroxidase [29]. Melatonin easily crosses cell membranes and leads to protection against free radical-induced damage to biomolecules [173].

Diabetic kidney is more sensitive to ischemia/reperfusion (I/R) injury correlated with enhanced oxidative stress. In a 4-week in vivo study, Shi et al. evaluated the therapeutic effects of melatonin on renal I/R of diabetic rats. Animals were treated with or without melatonin, and renal I/R injury was induced by clamping right and left renal pedicles for 30 min followed by reperfusion for 48 h. Diabetic rats treated with melatonin were protected against renal injury, in aspects of oxidative stress and cell apoptosis in kidney [174].

In accordance with the findings of Parving et al. [175], Motawi and colleagues indicating that the combination of melatonin with Losartan Potassium considerably ameliorates the urine creatinine and serum uric acid levels as well as kidney injury molecule (KIM-1) level, as glomerular damage index [176]. In addition, these agents decrease diabetes mellitus effects on the level of oxidative stress biomarkers, where melatonin and LSP combination significantly ameliorate the levels of GSH, SOD, MDA, and NO in rats with diabetic nephropathy [176]. Aberrant angiogenesis has a pathological role in diabetic nephropathy; however, endothelial-to-mesenchymal transition of glomerular endothelial cells results in enhanced permeability, leading to proteinuria [177, 178]. Melatonin inhibits endothelial-to-mesenchymal transition (EMT) in glomerular endothelial cells exposed to transforming growth factor-β2 (TGF-β2) and in the glomeruli of diabetic rats; this effect is mediated by the up-regulation of miR-497 expression leading to inhibited RhoA/Rho kinase (ROCK) activity [179].

Rashed and his colleagues have shown that incubation of mesenchymal stem cells with melatonin before administration resulted in the improvement of autophagy pathway, elevation of SOD1 level and further decline in TGF-β level in the kidney tissue of diabetic rats compared to those treated with mesenchymal stem cells alone [180].

Onk et al. [173] found enhanced IL-33 levels in the kidney tissue following contrast-induced nephropathy (CIN) in diabetic rats; administration of melatonin in these animals considerably attenuated kidney tissue concentrations of inflammatory cytokines, oxidative stress biomarkers, and IL-33, indicating potential roles of melatonin in the treatment of diabetic nephropathy. Melatonin also reduced serum creatinine in rats with diabetic nephropathy.

In an in vitro study, Ji et al. evaluated protective roles of melatonin against angiotensin II–mediated diabetic nephropathy. They revealed that melatonin markedly enhanced the proliferative rate of cells and decreased angiotensin II-mediated apoptosis, as shown by declined concentrations of apoptotic proteins expression, such as Bax and caspase-3. Also, the recovery of mitochondrial function and reduced oxidative stress were implicated in the protective effect of melatonin. Additionally, the inhibition of Janus kinase (JAK)-signal transducer and activator of transcription (STAT) pathway demonstrated that cytokine-induced inflammation is also targeted by melatonin [181].

Gumustekin et al. assessed the efficacy of melatonin in the treatment of rats with diabetic nephropathy. After 14 weeks of diabetes induction, 10 mg/kg melatonin was given to diabetic rats for 5 days. Melatonin could not improve hemorheological abnormalities, such as erythrocyte deformability and aggregation, but ameliorated renal injuries by suppressing apoptosis process [182].

It is obvious that melatonin affects various signaling pathways resulting in the reduction of diabetes-induced renal damages (Fig. 1d); however, further studies should be performed to find the exact mechanism of action of melatonin for diabetic nephropathy. Notably, it seems that melatonin deserves to be used in the human trials as a complementary treatment to assess its effectiveness in the treatment of patients with diabetic nephropathy. Herein, Table 5 summarizes in vivo and in vitro studies on the effect of melatonin on diabetic nephropathy.

**Table 5:** The application of melatonin for the treatment of diabetic nephropathy

Type of study		Route of administration	Treatment duration	Target	Effect(s)	Refs.
In vivo dose (animal)	In vitro concentration (cell type)					
50 mg/L (male Sprague–Dawley rats)	50 μM (human renal GEnCs)	Drinking water	4 weeks	ROCK1, ROCK2, miR-497	Melatonin attenuated Endothelial-to-mesenchymal transition of GEnCs through modulating miR-497/ROCK signaling	[179]
–	5 μM (mesenchymal stem cells)	–	24 h	Beclin-1	MSCs therapy considerably ameliorated the renal functions. Its impact was intensified by melatonin pre-incubation.	[180]
200 µg/kg (male ZDF rats)	–	Intraperitoneal	8 weeks	HSP70, Caspase3, TGF-β, KIM-1	Melatonin revealed potential effects on protecting rats from deleterious action of diabetic nephropathy followed by its combination with rowatinex.	[197]
–	0/1, 1 mM (mouse podocytes)	–	48 and 72 h	Bax/Bcl–2, Jak/STAT	Melatonin showed anti-apoptotic effects in AngII-induced podocyte injury	[181]
200 µg/kg/day (male Wistar strain albino rats)	–	Oral	60 days	HSP70 Caspase3, TGF-β KIM-1	The combination of melatonin and Losartan Potassium exerted the most potent effects on treating the deleterious action of diabetes on rat kidney	[176]
20 mg/kg/day (male Sprague–Dawley rats)	–	Intraperitoneal	7 days	MPX, IL-33 IL-1*β* IL-6	Suppression of IL-33 with melatonin yields therapeutic potentials in diabetic kidney disease with contrast-induced nephropathy	[173]
10 mg/kg (male Sprague–Dawley rats)	–	Intraperitoneal	4 weeks	SIRT1/Nrf2/HO-1 signaling	Antioxidants effects	[174]
20 mg/L (male Wistar strain albino rats)	–	Drinking water	4 weeks	NADPH oxidase (NOX)	Melatonin showed antioxidant and nephroprotective effects	[198]
10 mg/kg/day (male db/db mice)	–	Intraperitoneal	30 days	SOD, CAT, MDA	Antioxidant effects	[199]
–	25 μM (kidney cells of db/db mice)	–	ND	Mitochondrial complex (MC)-1 MC-3	Antioxidant effects	[200]
10 mg/kg/day (male Wistar rats)	–	Intraperitoneal	5 days	Blood glucose, HbA1c	Antioxidant effects Anti-apoptotic effects	[182]
0.02% of drinking water (male Sprague–Dawley rats)	–	Drinking water	4 weeks	TGF-β1, FN	Potential effects on early changes in diabetic kidney	[201]
200 μg/kg/day (male Sprague–Dawley rats)	–	Intraperitoneal	15 days	SOD, CAT, GST, NO	Antioxidants effects	[202]
10 mg/kg/day (male Sprague–Dawley rats)	–	Intraperitoneal	8 weeks	xanthine oxidase (XO) MDA, GSH-Px, SOD	Antioxidants effects	[203]
200 μg/kg/day (male Wistar strain albino rats)	–	Intraperitoneal	4 weeks	IGF-1, anti-laminin β1	Antioxidants effects	[204]

*ND* not defined

### Melatonin and diabetic wound healing

Diabetic wounds are leading cause of diabetes-associated amputations, resulting in poor quality of patients’ life and high medical cost [183]. It is characterized by impaired or diminished production of growth factor, granulation tissue quantity, epidermal barrier function, collagen accumulation, macrophage function, and angiogenic response [184, 185]. Disruption in healing process may result from ischemic conditions caused by intrinsic factors such as signaling abnormalities, neuropathy, and vascular problems as well as extrinsic factors including trauma and wound infection [186, 187]. Denervation with sensory abnormalities of the nerves further exacerbates the slowed microcirculation [188].

Melatonin, which is also produced in the skin [189], accelerates wound healing in full-thickness incisional wounds [190]. Ji e al. carried out an investigation to evaluate melatonin potential in improving diabetic wound healing and the possible mechanism. Treatment of mice bone marrow-derived endothelial progenitor cells (EPCs) with melatonin inhibits advanced AGE-mediated cellular dysfunction and apoptosis. Melatonin is reported to enhance autophagy flux characterized by the elevation of LC3 level, and reduction of p62 accumulation. Moreover, AMPK/mTOR signaling pathway is implicated in stimulating autophagy via melatonin. In a diabetic wound healing model, melatonin improved disrupted wound healing. Therefore, melatonin is suggested to improve disrupted wound healing through inhibiting apoptotic events by stimulation of autophagy pathway [191].

Melatonin also promotes umbilical cord blood (UCB)-mesenchymal stem cells (MSCs) motility through enhancing cytoskeletal reorganization which this effect is mediated by melatonin MT2 receptor triggering Gq protein alpha (Gαq)-dependent protein kinase (PK)Cζ phosphorylation regulating Focal adhesion kinase (FAK)/paxillin-mediated cell division cycle 42 (Cdc42)/actin-related proteins (Arp2/3) activation [192].

In keratinocytes, melatonin reduces high glucose-induced the release of inflammatory cytokines, such as interleukin (IL)-1β, IL-6, IL-8 and tumor necrosis factor-α associated with the reduction of ROS formation and the elevation of SOD activity. In diabetic wound, melatonin causes essential events in the healing process including suppression of apoptosis, promotion of migration and proliferation, reduction of oxidative stress, and suppression of inflammatory process in keratinocytes [193]. Hence, melatonin is suggested to be useful in diabetic foot ulcer through regulating keratinocyte activity. Table 6 summarizes investigations have evaluated the effect of melatonin on diabetic wound.

**Table 6:** Investigations on melatonin and diabetic wound healing

Type of study		Route of administration	Treatment duration	Targets	Effect(s)	Refs.
In vivo dose (animal)	In vitro concentration (cell type)					
1.2 mg/kg (male Sprague–Dawley rats)	–	Intra‐dermal	1 week	iNOS, COX‐1, COX‐2, VEGF, arginase‐I, arginase‐II, HO‐1 and HO‐2	Melatonin improved the quality of wound healing and scar formation	[190]
–	10, 20, 50, 100, and 200 μM (endothelial progenitor cells (EPCs))	–	2 h	mTOR, 4EBP1, AMPKα, p70S6K, and P62	Melatonin inhibited apoptosis and dysfunction of EPCs via autophagy flux stimulation	[191]
– (male ICR mice)	1 μm Umbilical cord blood (UCB)‐MSCs	24 h	FAK/paxillin, Cdc42/Arp2/3, PKC, Gαq and	–	Melatonin enhanced wound closure, granulation, and re‐epithelialization at mouse skin wound sites	[192]
–	1 mM keratinocytes	24 h	TNF-α, IL-1β, IL-6, IL-8, ROS, SOD, MDA	–	Melatonin increased migration and proliferation and reduced apoptosis of keratinocytes	[193]

### Melatonin application for diabetic patients in clinical practice

Raygan et al. carried out a randomized, double-blind clinical trial on diabetic patients with coronary heart disease. Sixty patients were divided into two groups to receive either placebo or 10 mg melatonin for 12 weeks. Melatonin had appropriate impacts on glycemic control, HDL-cholesterol, serum levels of high-sensitivity C-reactive protein (hs-CRP), blood pressure, plasma MDA, NO, and GSH as well as mental health parameters [194].

Kadhim et al. performed a clinical trial on patients with type 2 diabetes who were poorly controlled with metformin. Forty-six patients with type 2 diabetes were divided into three groups and were treated for 90 days with daily oral administration of melatonin (10 mg), zinc acetate (50 mg), or both supplements in addition to the routine used metformin or placebo. Daily intake of zinc and melatonin reduced the level of microalbuminuria and ameliorated the lipid profile. In conclusion, the addition of these supplements to metformin ameliorated the tissue responses to this oral hypoglycemic agent [67].

Another investigation was conducted to assess therapeutic efficacy of melatonin and zinc in glycemic control of type 2 diabetic patients poorly controlled with metformin. The supplements were administered at the same doses in 90 days. They revealed that the combination of zinc acetate and melatonin, when utilizes alone or in combination with metformin, ameliorates fasting and post-prandial glucose levels in diabetic subjects [195]. Regarding what discussed melatonin supplementations could be used for diabetic complications in human studies. To prove the effectiveness and safety of melatonin as a complementary treatment for diabetic patients, it is important to design large clinical trials.

## Conclusions

Due to the rapid increase of the global DM prevalence, exploring efficient therapies for diabetic complications is an important issue. Routine treatments for DM are generally based on metabolic control, and existing drugs cannot totally control the development of complications. Researches have recently indicated the capability of melatonin to modulate various signaling pathways and demonstrated its therapeutic and preventive effects in several diseases, such as DM. Preclinical and clinical studies have shown different mechanisms of action of melatonin including reduction of glucose production, and improvement of pro-inflammatory pathways as well as oxidative stress state in DM patients. Previously, it has been revealed that melatonin treatment ameliorates DM through regulating the lipid and glucose metabolism, reducing the insulin resistance and increasing the sensitivity to insulin in experimental models of DM. It will be crucial to perform human clinical trials utilizing combinations of melatonin with current therapeutic agents for treatment and prevention of cardiomyopathy, neuropathy, retinopathy, nephropathy, and other diabetic complications.

## Data Availability

Not applicable
